# A machine learning model based on clinical features and ultrasound radiomics features for pancreatic tumor classification

**DOI:** 10.3389/fendo.2024.1381822

**Published:** 2024-06-17

**Authors:** Shunhan Yao, Dunwei Yao, Yuanxiang Huang, Shanyu Qin, Qingfeng Chen

**Affiliations:** ^1^ Medical College, Guangxi University, Nanning, China; ^2^ Monash Biomedicine Discovery Institute, Monash University, Melbourne, VIC, Australia; ^3^ Department of Gastroenterology, The First Affiliated Hospital of Guangxi Medical University, Nanning, China; ^4^ Department of Gastroenterology, The People’s Hospital of Baise, Baise, China; ^5^ School of Computer, Electronic and Information, Guangxi University, Nanning, China

**Keywords:** pancreatic tumors, malignant, clinical features, radiomics features, machine learning, classification nomogram

## Abstract

**Objective:**

This study aimed to construct a machine learning model using clinical variables and ultrasound radiomics features for the prediction of the benign or malignant nature of pancreatic tumors.

**Methods:**

242 pancreatic tumor patients who were hospitalized at the First Affiliated Hospital of Guangxi Medical University between January 2020 and June 2023 were included in this retrospective study. The patients were randomly divided into a training cohort (n=169) and a test cohort (n=73). We collected 28 clinical features from the patients. Concurrently, 306 radiomics features were extracted from the ultrasound images of the patients’ tumors. Initially, a clinical model was constructed using the logistic regression algorithm. Subsequently, radiomics models were built using SVM, random forest, XGBoost, and KNN algorithms. Finally, we combined clinical features with a new feature RAD prob calculated by applying radiomics model to construct a fusion model, and developed a nomogram based on the fusion model.

**Results:**

The performance of the fusion model surpassed that of both the clinical and radiomics models. In the training cohort, the fusion model achieved an AUC of 0.978 (95% CI: 0.96–0.99) during 5-fold cross-validation and an AUC of 0.925 (95% CI: 0.86–0.98) in the test cohort. Calibration curve and decision curve analyses demonstrated that the nomogram constructed from the fusion model has high accuracy and clinical utility.

**Conclusion:**

The fusion model containing clinical and ultrasound radiomics features showed excellent performance in predicting the benign or malignant nature of pancreatic tumors.

## 1 Introduction

Pancreatic cancer is one of the major causes of cancer related deaths in developed countries and one of the most lethal malignant tumors in the world (1). Currently, pancreatic cancer has surpassed breast cancer to become the third leading cause of cancer-related deaths in the United States, and it is projected to become the second leading cause, after only lung cancer, of cancer-related mortality before 2040 (2, 3). The high mortality rate of pancreatic cancer primarily stems from the fact that patients are often diagnosed at a late stage of the disease, missing the optimal window for effective treatment (4). This situation is compounded by the lack of a single attributable risk factor for pancreatic cancer and the pancreas’ anatomically difficult-to-access location, which impedes routine disease screening. Additionally, early-stage pancreatic cancer typically presents with no symptoms or with nonspecific symptoms, and the absence of diagnostic biomarkers for early-stage tumors further limits early detection (5). The incidence of pancreatic cancer is rising due to a variety of factors, including the obesity epidemic and increased life expectancy (2, 6). The development of new diagnostic strategies is crucial for enhancing treatment decision-making and assessing patient prognosis.

In recent years, with the continual development of artificial intelligence technologies, an increasing number of researchers have begun to explore their application in the diagnosis of pancreatic cancer. At present, the application of artificial intelligence technologies in the genome and transcriptome analysis of pancreatic cancer is relatively mature. For instance, Biao Zhang and colleagues have developed a pancreatic cancer survival prediction model using random forest algorithms and Cox regression analysis, integrating single-cell and bulk RNA sequencing (7). They have also employed eight different AI algorithms to assess the immunological microenvironment features of pancreatic cancer, providing new biomarkers for its diagnosis and treatment (8). However, there is still a shortage of studies on the AI-assisted diagnosis of pancreatic cancer based on clinical and radiological features. Currently, some researchers have used deep learning techniques to analyze pancreatic CT images and have successfully identified pancreatic cancer lesions (9). Other researchers have also utilized clinical variables to construct mathematical models and scoring systems for assessing the risk of pancreatic cancer (10). These methods have shown varying degrees of effectiveness in clinical trials. However, the use of only clinical features or radiomics alone has been found to be insufficient for reliable differential diagnosis (11–14). The development of mathematical models and scoring systems that combine clinical and radiomics features could improve diagnostic accuracy (15). Yu Hu and colleagues developed a model that integrates clinical risk factors and ultrasound features for predicting the malignancy of soft tissue tumors in the limbs (16). Amogh and colleagues applied deep learning to combine clinical variables with MRI data to construct a classification model for prostate cancer (17). These models have achieved high diagnostic performance, indicating that the synergistic use of clinical and radiomics features in model construction can enhance the diagnostic capabilities of the models.

This study aimed to integrate clinical and ultrasound radiomics features of pancreatic tumor patients to construct a fusion model that can accurately predict the benign or malignant nature of pancreatic tumors.

## 2 Materials and methods

### 2.1 Patient enrollment

The ethics review board of The First Affiliated Hospital of Guangxi Medical University approved this retrospective study and waived the requirement for written informed consent. The inclusion criteria for patients were as follows: diagnosed with either benign or malignant pancreatic tumors via pathological examination and treated at the First Affiliated Hospital of Guangxi Medical University from January 2020 to June 2023. The exclusion criteria for patients were as follows: (1) had received any antitumor treatment prior to laboratory tests or ultrasonography. (2) Patients with malignant tumors in other parts of the body. (3) Patients with recurrent pancreatic tumors. (4) Patients with incomplete clinical or ultrasound data. A total of 242 eligible patients were randomly divided into two cohorts at a 7:3 ratio: the training cohort (n=169) and the test cohort (n=73).

### 2.2 Clinical data collection

All patients’ clinical data, ultrasound images, and pathological examination results were sourced from the hospital’s Health Information System (HIS). The clinical data included general patient information, clinical signs, and laboratory test results. General information included sex, age, and body mass index (BMI). Clinical signs included abdominal pain, jaundice, tumor location, tumor diameter, and tumor subtype. The laboratory test results included blood type, blood sugar, total bilirubin, low-density lipoprotein cholesterol, apolipoprotein B, CA125, CA199, and other parameters. During data preprocessing, features with more than 10% missing values were discarded. For continuous numerical variables, missing values were imputed using the median. For categorical variables, the mode was employed to fill in missing values. Continuous variables such as BMI, CA125 and CA199 were converted into categorical variables according to the criteria of the WHO classification. Ultimately, a complete set of 28 clinical features was obtained.

### 2.3 Radiomics feature collection

All the procedures were performed according to the Image Biomarker Standardization Initiative (IBSI) standards. Ultrasound images were acquired by Olympus or Fuji ultrasonic equipment with linear probes of 5.0–7.5 MHz and saved in DICOM format for further analysis. Echo texture analysis of the ultrasound images was conducted using a computer program specifically designed for ultrasound image analysis (MaZda v4.6; Institute of Electronics, Technical University of Lodz, Poland) (18).

Endoscopic ultrasonography was performed on patients by gastroenterologists with 10 years of experience, and 1 image containing the tumor was collected for each patient for feature extraction. The ultrasound grayscale images were imported into MaZda software, where a physician with ten years of experience delineated the region of interest (ROI) encompassing the tumor on the images. Subsequently, MaZda software was used to extract the features of the ROIs. Then, to measure interobserver reproducibility, a total of 100 images were randomly selected to be re-segmented by a senior radiologist with 20 years of experience. An interclass correlation coefficient (ICC)>0.75 indicates high feature stability. Discrepancies were resolved through consultation. The radiologists were unaware of the specific histopathological type when delineating the ROI.

**Figure 1:**
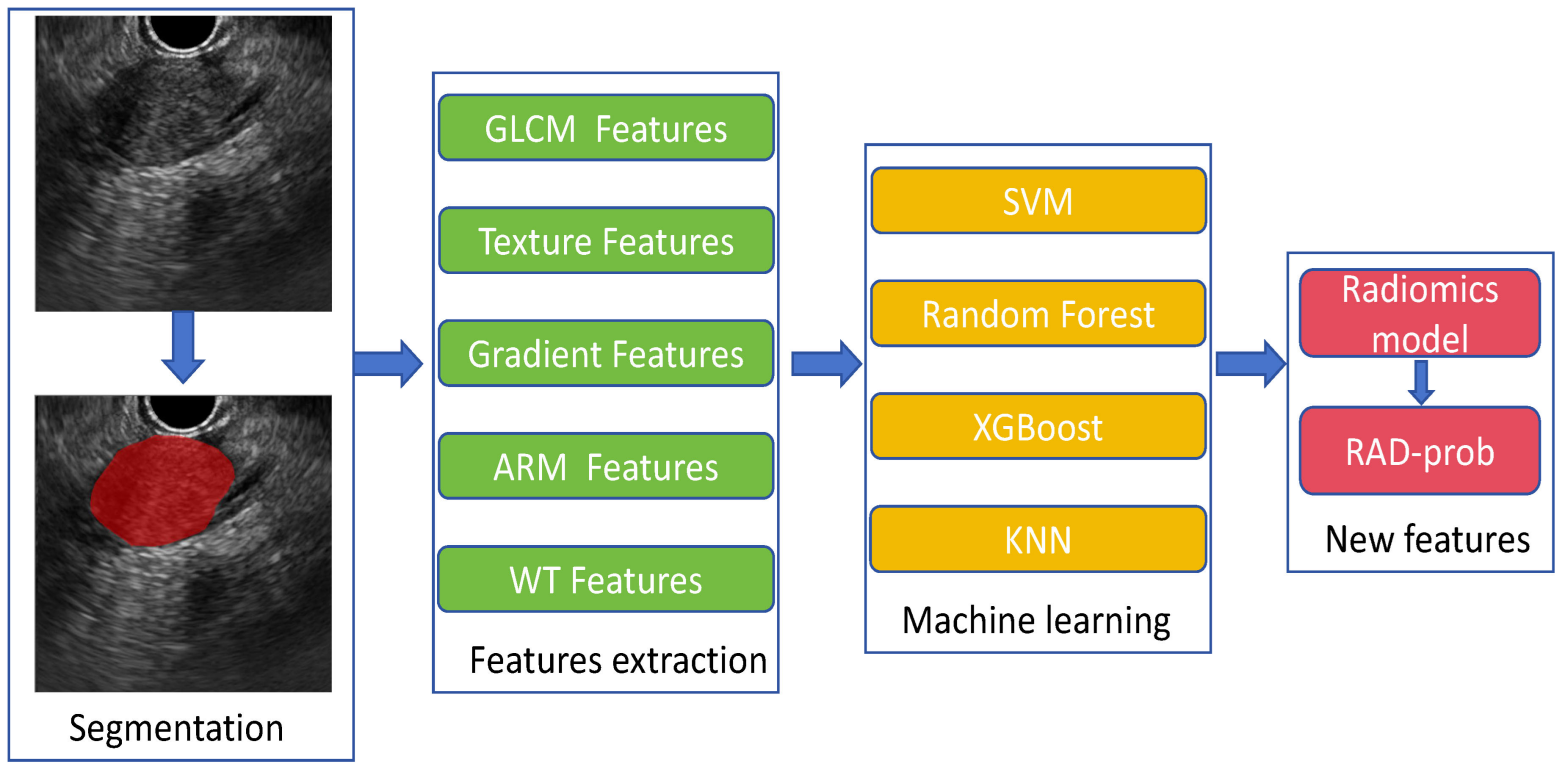
Extraction and modeling of radiomics features.

A total of 306 features were extracted and categorized into seven major types: (1) first-order statistics; (2) shape-based; (3) gray level co-occurrence matrix (GLCM); (4) gray level run length matrix (GLRLM); (5) gray level size zone matrix (GLSZM); (6) neighboring gray tone difference matrix (NGTDM); (7) gray level dependence matrix (GLDM). Feature preprocessing included handling outliers through log transformation and normalization using min–max scaling. Following these steps, we obtained a radiomics feature dataset for subsequent analysis from 242 patients. The process of radiomics feature extraction and modeling is illustrated in **Figure 1**.

### 2.4 Model establishment

Clinical Model: We utilized 28 candidate clinical features to construct the clinical model. In the training cohort, both univariate and multivariate logistic regression analyses were employed to compare the differences in clinical features between patients with benign and malignant pancreatic tumors. Features with significant differences (p < 0.05) were selected to establish the clinical model.

Radiomics Model: For the radiomics model, 306 candidate radiomics features were used. In the training cohort, four machine learning algorithms—support vector machine (SVM), random forest, XGBoost, and K-nearest neighbors (KNN) —were utilized to construct classification models, and the best-performing model was chosen as the radiomics model.

**Figure 2:**
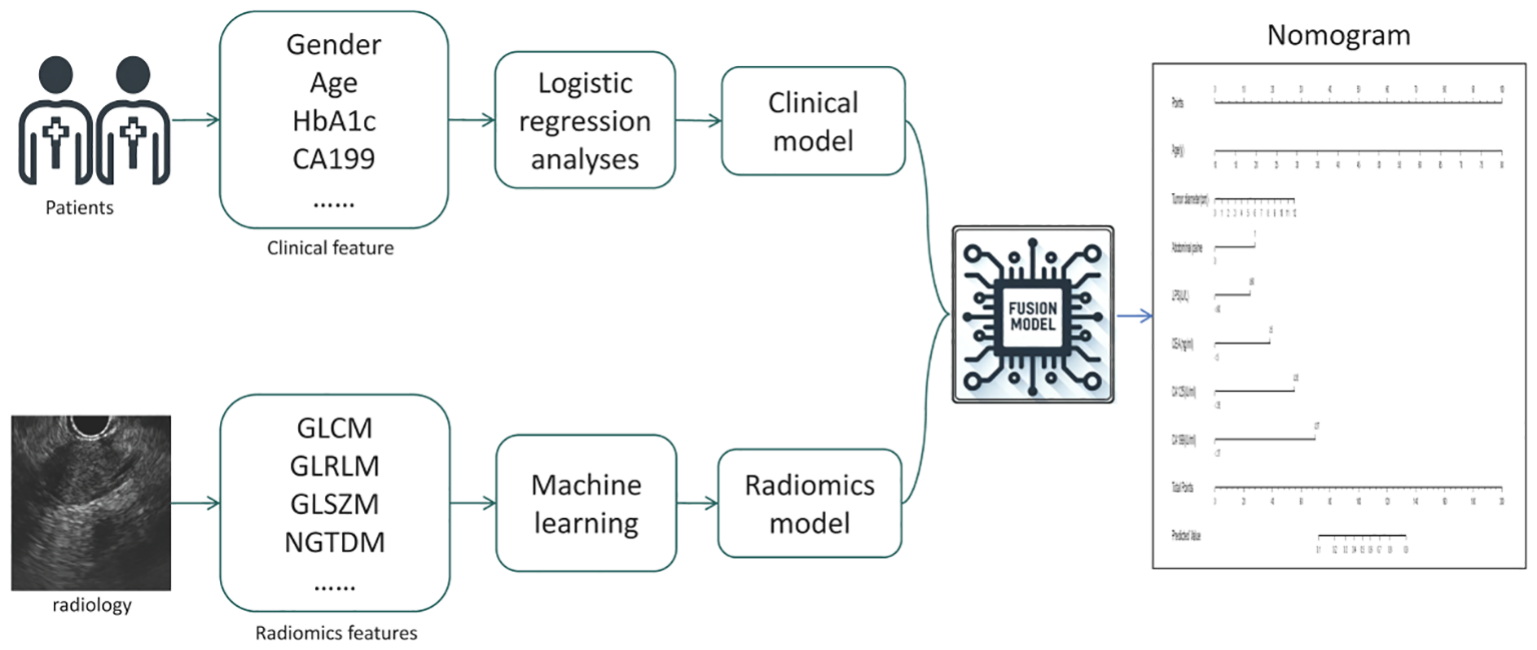
Flowchart of the Model Construction Process.

Fusion Model: We calculated the probability of each patient developing a malignant pancreatic tumor using the radiomics model, and named this probability the RDA-prob. A fusion model was constructed through multivariate logistic regression analysis by combining RDA-prob data with clinical data. Based on the fusion model, a nomogram was developed. The process of model construction is illustrated in **Figure 2**.

### 2.5 Statistical analysis

Statistical analysis was performed using Python software (version 3.12), R software (version 4.0.2), and SPSS software (version 26.0). For binary categorical variables, the chi-square test was utilized; for continuous variables, comparisons between the two groups were performed using the independent samples t-test or the Wilcoxon rank sum test, depending on the data distribution. A two-tailed P-value < 0.05 was considered to indicate statistical significance. The performance metrics for the models included accuracy, precision, recall, F1 score, and area under the curve (AUC) value, along with the generation of ROC curves. The model’s performance was evaluated on the training cohort using 5-fold cross-validation and was also calculated on the test cohort. The performance of the nomogram was assessed through calibration curves and decision curves.

## 3 Results

### 3.1 Study population and baseline characteristics

A total of 242 patients with pancreatic tumors were randomly divided at a 7:3 ratio into a training cohort (n = 169) and a test cohort (n = 73). There were no statistically significant differences in basic clinical characteristics, such as sex, age, BMI, abdominal pain, jaundice, tumor diameter, or tumor classification (P > 0.05), between the training and test cohorts. The basic clinical characteristics of the patients are shown in **Table 1**.

**Table 1:** Baseline clinical information of patients.

Variable	Training cohort(n=169)	Test cohort(n=73)	P-Value
Male/female	91/78	42/31	0.709
Age	56.84 ± 13.40	58.12 ± 11.67	0.454
BMI	21.90 ± 3.57	21.97 ± 3.06	0.879
abdominal pain	126	53	0.751
Jaundice	35	14	0.785
Pancreatic head	104	47	0.675
Pancreatic body	40	15	0.595
Pancreas tail	34	14	0.866
Tumor diameter	4.04 ± 2.04	3.59 ± 1.72	0.081
Malignant tumor	117	52	0.755
Benign tumor	52	21	0.755

### 3.2 Clinical model construction and evaluation

**Figure 3:**
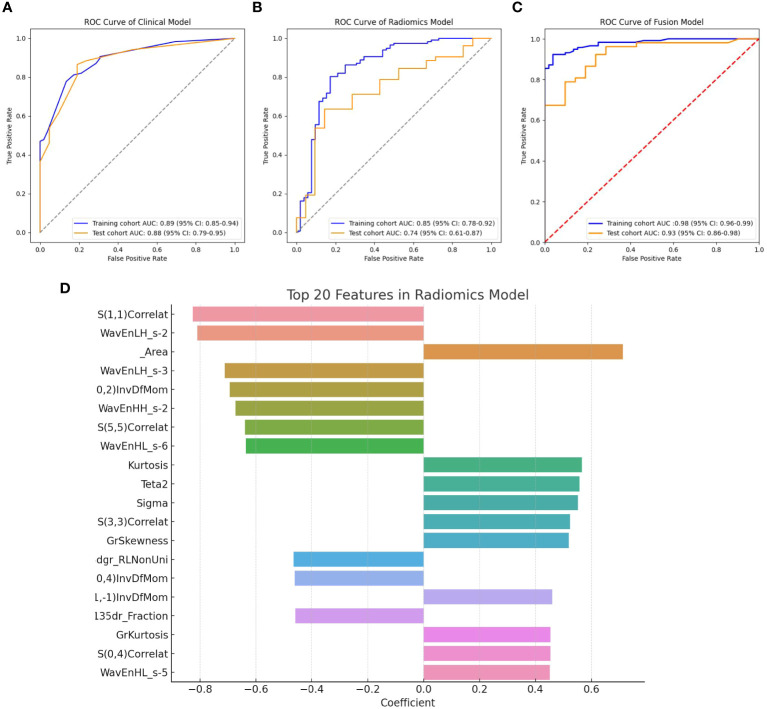
**(A)** ROC curve for the clinical model; **(B)** ROC curve for the radiomics model; **(C)** ROC curve for the fusion model; **(D)** Top 20 primary features in the radiomics model.

In the training cohort, univariate and multivariate logistic regression analyses were conducted on 28 clinical features, revealing that age, abdominal pain, CEA, CA125, CA199, and HbA1c were significant independent predictors. Therefore, these factors were used to construct the classification model. Although the P-value for tumor diameter was slightly above 0.05 (0.058), it was still close to the level of significance. Given its clinical importance, tumor diameter was also included as a feature in the clinical model. The clinical model achieved an AUC of 0.892 (95% CI: 0.85–0.94) in 5-fold cross-validation. In the test cohort, the AUC was 0.882 (95% CI: 0.74–0.95). The results of univariate and multivariate logistic regression analyses in the training cohort are shown in **Table 2**. The detailed results of the clinical model’s performance are presented in **Table 3**. The ROC curve is illustrated in **Figure 3A**.

**Table 2:** Results of the logistic regression analysis in the Training cohort.

Feature	Univariate analysis OR (95%CI)	p-value	Multivariate analysis OR (95%CI)	p-value
Sex	0.89 [1.47, 3.89]	0.738	–	–
Age	1.11 [0.00, 0.05]	0.000	1.12 [1.08, 1.17]	0.000
BMI	0.91 [0.83, 1.00]	0.046	0.95 [0.84, 1.06]	0.353
Abdominal pain	4.42 [2.12, 9.21]	0.000	4.32 [1.61, 11.59]	0.004
Jaundice	2.02 [1.38, 2.83]	0.126	–	–
Pancreatic head	1.12 [1.25, 3.52]	0.732	–	–
Pancreatic body	1.45 [1.43, 2.99]	0.367	–	–
Pancreas tail	0.77 [1.64, 3.44]	0.523	–	–
Tumor diameter	1.26 [0.43, 1.98]	0.016	1.24 [0.99, 1.55]	0.058
Blood type A	1.01 [0.49, 2.08]	0.977	–	–
Blood type B	1.20 [0.53, 2.71]	0.661	–	–
Blood type AB	–	1.000	–	–
Blood type O	0.63 [0.32, 1.23]	0.176	–	–
D-II	1.96 [0.43, 4.45]	0.109	–	–
HbA1c	3.07 [0.86, 6.24]	0.002	3.44 [1.22, 9.68]	0.02
BS	1.28 [0.61, 2.69]	0.514	–	–
T-BIL	1.89 0.92, 3.86]	0.081	–	–
D-BIL	2.32 [1.12, 4.79]	0.023	2.51 [0.52, 12.14]	0.25
ALP	2.17 [1.06, 4.43]	0.033	0.36 [0.07, 1.76]	0.21
T-CHO	1.08 [0.51, 2.29]	0.838	–	–
TG	0.93 [0.45, 1.92]	0.841	–	–
LDL-C	0.80 [0.40, 1.59]	0.531	–	–
apoB	0.82 [0.38, 1.76]	0.602	–	–
LPS	1.34 [0.61, 2.94]	0.459	–	–
AMS	0.64 [0.21, 1.90]	0.420	–	–
CEA	11.36 [3.35, 38.58]	0.000	4.99 [1.17, 21.28]	0.03
CA125	12.63 [4.28, 37.29]	0.000	10.31 [2.73, 38.91]	0.00
CA199	10.81 [4.93, 23.68]	0.000	12.91 [4.84, 34.41]	0.00

BMI, body mass index; D-II, D-Dimer; HbA1c, Glycated hemoglobin A1c; BS, Blood Sugar; T-BIL, Total Bilirubin; D-BIL, Direct Bilirubin; ALP, Alkaline Phosphatase; T-CHO, Total Cholesterol; TG, Triglycerides; LDL-C, Low-density lipoprotein cholesterol; apoB, Apolipoprotein B; LPS, Lipopolysaccharide; AMS, Amylase; CEA, Carcinoembryonic Antigen; CA125, Carbohydrate Antigen 125; CA199, Carbohydrate Antigen 19–9.

**Table 3:** Performance of the clinical model, radiomics model, and fusion model.

	Accuracy	Precision	Recall	F1 Score	AUC
5-CV					
clinical model	0.834	0.868	0.897	0.882	0.892
Radiomics model	0.811	0.824	0.923	0.871	0.854
Fusions model	0.917	0.940	0.940	0.940	0.978
Test cohort					
clinical model	0.849	0.918	0.865	0.891	0.882
Radiomics model	0.699	0.759	0.846	0.800	0.739
Fusions model	0.863	0.904	0.904	0.904	0.925

### 3.3 Radiomics model construction and evaluation

In the training cohort, classification models were constructed using 306 radiomics features with four different algorithms: SVM, random forest, XGBoost, and KNN. The performance of the model constructed using these four algorithms is shown in **Table 4**. Among them, the model built using the KNN algorithm performed the best and is identified as the radiomics model. The radiomics model achieved an AUC of 0.854 (95% CI: 0.78–0.92) in 5-fold cross-validation (5-CV), and an AUC of 0.739 (95% CI: 0.61–0.87) in the test cohort. The ROC curve for the radiomics model is illustrated in **Figure 3B**. The top 20 primary features of the radiomics model are shown in **Figure 3D**.

**Table 4:** Performance of radiation models constructed by different algorithms.

	Accuracy	Precision	Recall	F1 Score	AUC
5-CV					
SVM	0.740	0.759	0.923	0.831	0.780
Random Forest	0.734	0.776	0.873	0.819	0.780
XGBoost	0.752	0.801	0.855	0.827	0.798
KNN	0.811	0.824	0.923	0.871	0.854
Test cohort					
SVM	0.753	0.793	0.885	0.836	0.639
Random Forest	0.671	0.769	0.769	0.769	0.620
XGBoost	0.644	0.750	0.750	0.750	0.601
KNN	0.699	0.759	0.846	0.800	0.739

### 3.4 Fusion model construction and evaluation

The radiomics model was utilized to calculate the probability of each patient developing a malignant pancreatic tumor, and these probabilities were assigned to a new feature named the RDA-prob. By combining the RDA-prob with clinical data in a multivariate logistic regression analysis, four features were selected for constructing the fusion model: age, RDA-prob, CA125, and CA199. The fusion model achieved an AUC of 0.978 (95% CI: 0.96–0.99) in 5-fold cross-validation and an AUC of 0.925 (95% CI: 0.86–0.98) in the test cohort. The fusion model consistently outperformed the other two models in both the five-fold cross-validation and the test cohort, demonstrating its robustness and generalizability. The detailed results of the performance of the fusion model are provided in **Table 3**. The ROC curve for the fusion model is shown in **Figure 3C**.

### 3.5 Nomogram

**Figure 4:**
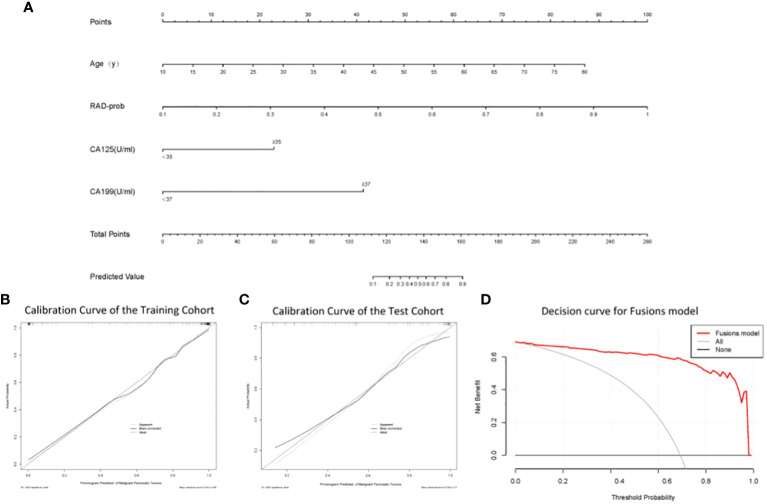
**(A)** The nomogram; **(B)** Calibration curve analysis of the nomogram in the training cohort; **(C)** Calibration curve analysis of the nomogram in the test cohort; **(D)** Decision curve analysis of the nomogram.

A nomogram was constructed based on the variables selected by the fusion model (**Figure 4A**). This nomogram provides a visual method for measuring the results of the fusion model, allowing for the rapid calculation of predictive probabilities for individual patients. To use the nomogram, one must first determine the position of each variable on its respective axis and then draw a line to the points axis to obtain the score for that variable. The scores for all variables are summed to obtain a total score, which is then located on the total points axis. The probability corresponding to this total score is the likelihood of the pancreatic tumor being malignant. Calibration curves (**Figures 4B, C**) demonstrate that the nomogram has good accuracy in both the training and test cohorts. The decision curve (**Figure 4D**) indicates that the nomogram has high clinical utility.

## 4 Discussion

In our study, we discovered that age, abdominal pain, tumor diameter, CA199, CEA, CA125, LPS, and HbA1c are independent predictors of malignant pancreatic tumors. These findings align with those of other studies (19–25). Several studies have shown a positive correlation between age, abdominal pain, tumor diameter and the probability of predicting malignant pancreatic tumors (19, 20). CA199 is the only serum biomarker for pancreatic cancer recommended in the European Oncology Guidelines, yet its sensitivity and specificity are approximately 80%, indicating limited diagnostic performance (21). Like CA199, CA125 is a high-molecular-weight glycoprotein that is currently primarily used as an auxiliary diagnostic tool for ovarian cancer (22). Several studies have shown a positive correlation between age, abdominal pain, tumor diameter, and the probability of predicting malignant pancreatic tumors (23). CEA, LPS, and HbA1c have also been confirmed by multiple studies to be associated with pancreatic cancer (24, 25). The results of our study support these findings, and based on the identified independent predictors, we constructed a clinical classification model. This model demonstrated good classification performance and exhibited robustness in both the training cohort and the test cohort.

Subsequently, our study analyzed endoscopic ultrasound images of patients with pancreatic tumors. Radiographic images can provide quantitative and reproducible texture information (26), and by extracting and quantifying texture features within images, significant reference information can be provided for the diagnosis, treatment, and prognosis, with the potential to enhance the diagnostic performance of models. Recently, several papers have been published by scholars on the application of image texture features in patients with pancreatic lesions. Zixing Huang and colleagues compared CT image texture features of patients with pancreatic lymphoma and pancreatic adenocarcinoma and identified 60 texture features with significant differences (27). Xudong Li and others analyzed MRI images of 119 pancreatic tumor patients and found four texture features that could provide information for tumor differentiation (28). These research results offer promise for the development of new radiological biomarkers.

In our study, we extracted 306 radiomics features from endoscopic ultrasound images of pancreatic tumors and constructed radiomic classification models using four different machine learning algorithms to compare their performance. The results showed that the model based on the KNN algorithm performed optimally, hence it was chosen as the primary radiomic model. Given the difficulty in interpreting the association between individual ultrasound radiomics features and potential pathological characteristics, we adopted the research methodologies of Fei Yao (29) and Weichen Zhang (30), integrating multiple ultrasonic radiomics features into a multi-element parameter, RAD-prob, to simplify the complexity of multi-feature analysis. The radiomics features used to construct RAD-prob range from basic morphological characteristics to advanced texture features, revealing the variability, heterogeneity, and inconsistency of the lesions. For instance, Gray Level Co-occurrence Matrix (GLCM) features such as S(1,1) Correlat and S(5,5) Correlat demonstrate the correlation between pixels in different directions; wavelet transform energy features like WavEnLH_s-2 and WavEnHH_s-2 reveal high-frequency textures and edge details of the image; first-order statistical features such as Area and Kurtosis describe the size of the image region and the peak characteristics of the gray level distribution; and inverse difference moment features like 0,2) InvDfMom indicate the uniformity and local similarity of the image texture. These comprehensive feature analyses not only exhibit the physical and geometrical attributes of pancreatic cancer but also highlight key biological markers in the ultrasound images, providing valuable insights into the structure and tissue characteristics of the lesion area, thereby supporting clinical diagnosis and treatment decisions.

To date, no single radiological or laboratory test has been able to reliably distinguish between malignant and nonmalignant pancreatic tumors (31). The preliminary diagnosis of pancreatic tumors depends on a comprehensive analysis of clinical features, laboratory tests, and radiological examinations rather than a single parameter (32). Traditional diagnostic methods, which rely on doctors interpreting test reports, are limited by the individual experiences of the doctors, potentially leading to inconsistent diagnoses (33). Artificial intelligence-based diagnostic models can objectively assist in diagnosis by utilizing a variety of different parameters (33, 34). Qian Lu and colleagues developed a multimodal model that integrated CT features and clinical characteristics, which performed better in predicting lymph node metastasis in pancreatic cancer than single-modality models (35). Wenjie Liang and colleagues developed and validated a nomogram model that combines radiomics features and clinical characteristics and showed excellent performance in differentiating the pathological grade of pancreatic neuroendocrine tumors (36). In our study, we also constructed a classification model that integrates clinical and radiomics features. We first used the radiomics model to calculate a new radiomics feature named RAD-prob and then combined the RAD-prob with clinical features to construct a fusion model through logistic regression. The performance of the fusion model surpassed that of both the clinical and radiomics models. In the training cohort, the combined model achieved an AUC of 0.978 (95% CI: 0.96–0.999) during 5-fold cross-validation. In the test cohort, the AUC was 0.925 (95% CI: 0.86–0.98).

Our fusion model offers a promising tool to assist doctors in making diagnostic decisions. To facilitate the clinical application of the model, we developed a nomogram based on the model parameters. This nomogram can quickly calculate predictive probabilities for individual patients, which may lead to its widespread use. Although the model developed in this study achieved good predictive results, there is still room for further optimization. In the future, methods such as ensemble learning and deep learning could be employed to further enhance the model’s predictive performance. Additionally, validation with multicenter data is needed to assess the model’s performance in real-world settings.

## 5 Conclusion

In summary, this study constructed three pancreatic tumor classification models using clinical and ultrasound radiomics features, each capable of differentiating between benign and malignant pancreatic tumors. The fusion model, which includes both clinical and ultrasound radiomics features, demonstrated exceptional performance in predicting the benign or malignant nature of pancreatic tumors. The nomogram of the fusion model, as a visual and personalized tool, can assist doctors in accurately identifying the type of pancreatic tumor.

## Data availability statement

The data supporting this study’s findings are available from the corresponding author upon reasonable request.

## Ethics statement

The studies involving humans were approved by The Ethics Review Board of The First Affiliated Hospital of Guangxi Medical University. The studies were conducted in accordance with the local legislation and institutional requirements. Written informed consent for participation was not required from the participants or the participants’ legal guardians/next of kin in accordance with the national legislation and institutional requirements.

## Author contributions

SY: Data curation, Formal analysis, Methodology, Writing – original draft. DY: Data curation, Methodology, Writing – original draft. YH: Methodology, Writing – original draft. SQ: Conceptualization, Data curation, Writing – review & editing. QC: Conceptualization, Writing – review & editing.
